# Raman chemical imaging, a new tool in kidney stone structure analysis: Case-study and comparison to Fourier Transform Infrared spectroscopy

**DOI:** 10.1371/journal.pone.0201460

**Published:** 2018-08-03

**Authors:** Vincent Castiglione, Pierre-Yves Sacré, Etienne Cavalier, Philippe Hubert, Romy Gadisseur, Eric Ziemons

**Affiliations:** 1 Department of Clinical Chemistry, CHU of Liège, University of Liège, Liège, Belgium; 2 University of Liege (ULiege), CIRM, VibraSante Hub, Laboratory of Pharmaceutical Analytical Chemistry, Liège, Belgium; University of Colorado Denver School of Medicine, UNITED STATES

## Abstract

**Background and objectives:**

The kidney stone’s structure might provide clinical information in addition to the stone composition. The Raman chemical imaging is a technology used for the production of two-dimension maps of the constituents' distribution in samples. We aimed at determining the use of Raman chemical imaging in urinary stone analysis.

**Material and methods:**

Fourteen calculi were analyzed by Raman chemical imaging using a confocal Raman microspectrophotometer. They were selected according to their heterogeneous composition and morphology. Raman chemical imaging was performed on the whole section of stones. Once acquired, the data were baseline corrected and analyzed by MCR-ALS. Results were then compared to the spectra obtained by Fourier Transform Infrared spectroscopy.

**Results:**

Raman chemical imaging succeeded in identifying almost all the chemical components of each sample, including monohydrate and dihydrate calcium oxalate, anhydrous and dihydrate uric acid, apatite, struvite, brushite, and rare chemicals like whitlockite, ammonium urate and drugs. However, proteins couldn't be detected because of the huge autofluorescence background and the small concentration of these poor Raman scatterers. Carbapatite and calcium oxalate were correctly detected even when they represented less than 5 percent of the whole stones. Moreover, Raman chemical imaging provided the distribution of components within the stones: nuclei were accurately identified, as well as thin layers of other components. Conversion of dihydrate to monohydrate calcium oxalate was correctly observed in the centre of one sample. The calcium oxalate monohydrate had different Raman spectra according to its localization.

**Conclusion:**

Raman chemical imaging showed a good accuracy in comparison with infrared spectroscopy in identifying components of kidney stones. This analysis was also useful in determining the organization of components within stones, which help locating constituents in low quantity, such as nuclei. However, this analysis is time-consuming, making it more suitable for research studies rather than routine analysis.

## Introduction

Urolithiasis is a spreading condition in developed countries, with an increasing prevalence from 5% to 8% between 1994 and 2010 [1,2]. Moreover, the recurrence rate is close to 30% at 5 years and even more in cystine or brushite stone-formers [3–5]. In addition, complications and highly active genetic urolithiasis can lead to chronic kidney disease [6]. Therefore, urological and preventive management of these patients is leading to great costs for health care systems. Furthermore, the recommendations for stone-formers treatment is highly dependent on the stone composition and biological evaluation [7]. Overall, acquired factors like diet, pathologies or even medication, together with inherited factors as sex, age, urologic malformation and genetics, have been associated to different stone compositions [8,9]. Moreover, in some specific and acute nephrolithiasis like cystinuria and adenine phosphoribosyltransferase deficiency, stone analysis is often the fastest and most reliable way to diagnosis [10,11]. This is why stone analysis is mandatory for every recurrent stone-formers [12,13].

Wet chemical analysis has been forsaken since many years as many valuable methods have been developed for stone composition determination. The Fourier Transform Infrared spectroscopy (FTIR) and powder X-Ray diffraction are considered as gold standard [14]. Since 1983, Raman spectroscopy, another vibrational method of analysis, has been tested in urolithiasis determination [15,16]. Notably, Daudon et al already assessed a large range of stone’s compounds [16]. Since, several studies used Raman spectroscopy in order to analyze urinary calculi [17–25]. It has been shown to be an adequate method in discriminating various chemical components of human kidney stone, and Raman spectroscopy was judged by most of these papers as complementary or comparable to FTIR analysis. Still, some other uncommon chemicals, mainly drugs, haven’t been tested yet. In addition, in most of these studies, even recent ones, Raman spectroscopy was only used as an alternative to FTIR on small sets of samples [20–22,24,25]. These studies used a destructive pathway without providing any structural information about the samples. Yet, chemical composition is not the only characteristic to take in consideration when analyzing kidney stones. Structure of stones as well as the presence of some particular elements, like Randall’s Plaque, are also providing information about stone formation and associated diseases [26–28]. Most of the time, calculi are made up of more than a single element, and the localization of the different chemical elements also gives clues on the stone “history” [29,30].

Raman microscopy has been widely tested in other human biological samples, especially in cancer diagnosis, where histological classification is mandatory for the diagnosis and treatment [31,32]. Some studies combined chemical and biological information to morphological and distribution parameters in order to correctly identify tumors or subcellular organites with high accuracy, sometimes even better than classical histology [31].

Raman has never been tested for the mapping of urolithiasis, this is why we aimed to use Raman chemical imaging (RCI) in order to perform an analysis of stone structure. The combination of Raman spectroscopy to microscopy enables the possibility to obtain distribution maps of the constituents in samples together with their spectra enabling their chemical characterization. This provides the localization of constituents within the stones, as it has been used in tumor mapping or in drug formulation control [33,34]. Thus, RCI should give clinically relevant information about lithogenesis. We sought to evaluate the utility of this method in advanced or routine analysis of stones with mixed composition.

## Material and methods

The Urolithiasis Laboratory of the CHU de Liège (Belgium), daily receives stones from in and outpatients of the hospital for FTIR analysis. After completion of the analysis, the remnant stones are anonymized and classified according to their composition and morphology. For this non interventional study, we selected 14 urinary stones according to their various composition obtained by FTIR. For instance, a calculus made up of calcium oxalate dihydrate and calcium oxalate monohydrate was selected because of these components may be difficult to discriminate by FTIR when they are mixed in unequal quantity [35]. Furthermore, some stones were chosen because of their heterogenous morphology (samples 10 and 11). Finally, we tested three samples containing drug deposits (samples 12 to 14). No author had access to patient’s information during the study. This study is in accordance with the Declaration of Helsinki and with our institutional review board «Comité d’Ethique Hospitalo-Facultaire de l’Université de Liège». The study was not specifically approved by the review board, and informed consent was not required because our ethical committee allow using samples remnants for methods comparison purpose while respecting patient’s anonymity according to the procedure of 23^th^ September 2014. None of the authors had access to the patients’ information by any means in this study.

### Fourier Transform Infrared spectroscopy

The samples were dried and conserved at room temperature before analysis. Calculi were first examined with a microscope (magnification 64x) for structure description. Stones were sliced in two equal parts in order to observe their section and nucleus. One half of the stone was analyzed by FTIR while the other part was studied by RCI. We didn’t analyze the same part of the stone with both analyses because patients’ follow-up requires quick FTIR results, while RCI analysis is slower. Selected fragments were ground in an agate mortar to realize pellets for the FTIR analysis. Potassium bromide was added. The final powder was homogenized and then passed into a press to make 3mm diameter pellets. Spectra were recorded between 4000 and 400 cm^-1^ (resolution 2cm^-1^) for 120 scans with Alpha FT-IR spectrophotometer (Bruker, Germany), and compared to the OPUS reference library, version 6.5 (Bruker Optics Inc). Semi-quantitative results were recorded in percentage.

### Raman chemical imaging

Confocal Raman experiments were performed with a Labram HR Evolution (Horiba scientific, France) equipped with a two-dimensional EMCCD detector (1600 × 200 pixels’ sensor) (Andor Technology Ltd.), a Leica 50x LWD Fluotar (NA: 0.55) objective and a 785 nm laser (XTRA II, Toptica Photonics AG, Germany). The spectra were collected with the LabSpec 6 (Horiba Scientific) software. A topographical analysis of the lithiasis was also realized using the EasyNav package of the Labspec 6 software (Horiba Scientific).

First, the stones were sliced and milled before RCI analysis, in order to obtain a smooth surface. The spectra were acquired over the spectral range of 464–1853 cm^-1^ with a 300 gr/mm grating. The laser power was reduced to 35mW at sample to preserve sample from burning. Seven accumulations of 2 seconds and a confocal hole set at 200μm were used. A step size of 5 to 50μm was used according to samples.

For data processing, once acquired, the hyperspectral images were unfolded and the spectra were smoothed using the Savitzky-Golay algorithm (window size of 5) [36], baseline corrected using the Asymmetric Least Squares (λ: 10^5^; p: 10^−3^) algorithm [37]. Pure spectra of the general map were resolved using MCR-ALS with a non-negativity constraint on both C and S matrices [38,39]. All computations were realized with Matlab R2016a (version 9.0, The Matworks, USA) and the PLS Toolbox 8.2.1 (Eigenvector Research, USA). Finally, the results of RCI were compared to the FTIR ones.

## Results

The fourteen samples were analyzed by both methods; Raman chemical imaging and Fourier transform infrared spectroscopy. In each sample, RCI succeeded in identifying almost all components recorded by FTIR. Calcium oxalate monohydrate (COM) and dihydrate (COD), anhydrous uric acid and dihydrate, apatite, struvite (magnesium ammonium phosphate or triple phosphate), whitlockite, brushite and ammonium urate were detected in all stones that contained some according to FTIR (Table 1). Concerning drugs, the metabolite of Sulfamethoxazole, N-Acetyl-Sulfamethoxazole, and the Atazanavir were identified. In contrast, the traces of Triamterene and its sulfate and hydroxyl conjugate metabolites weren’t detected by RCI in sample 13. Similarly, proteins and glycosaminoglycans were not identified by RCI method. Most of the tested components accounted for more than 15% of stones constituents according to FTIR analysis. The COM was correctly identified by RCI in the sample 10, in which it represented only 5% of FTIR stone composition. The FTIR analysis identified 10% of anhydrous uric acid and COD in samples 5 and 9 respectively, which were correctly detected by RCI too. In addition, the RCI analysis revealed small amount of apatite in sample 13 that hadn’t been detected by FTIR.

**Table 1:** Fourier Transform Infrared spectroscopy and Raman chemical imaging samples’ composition.

Sample	Fourier transform infrared	Raman chemical imaging
1	Calcium oxalate monohydrate (100%)	Calcium oxalate monohydrate
2	Calcium oxalate monohydrate (60%) Ammonium urate (30%) Proteins (10%)	Calcium oxalate monohydrate Ammonium urate
3	Calcium oxalate dihydrate (80%) Calcium oxalate monohydrate (15%) Apatite (5%)	Calcium oxalate dihydrate Calcium oxalate monohydrate Apatite
4	Anhydrous uric acid (100%)	Anhydrous uric acid
5	Uric acid dihydrate (90%) Anhydrous uric acid (10%)	Uric acid dihydrate Anhydrous uric acid
6	Apatite (80%) Calcium oxalate monohydrate (20%)	Apatite Calcium oxalate monohydrate
7	Struvite (95%) Proteins (5%)	Struvite
8	Brushite (90%) Calcium oxalate dihydrate (10%)	Brushite Calcium oxalate dihydrate
9	Brushite (25%) Whitlockite (25%) Apatite (25%) Proteins (20%) Calcium oxalate dihydrate (5%)	Brushite Whitlockite Apatite Calcium oxalate dihydrate
10	Apatite (40%) Struvite (35%) Ammonium urate (20%) Calcium oxalate monohydrate (5%)	Apatite Struvite Ammonium urate Calcium oxalate monohydrate
11	Apatite (30%) Calcium oxalate dihydrate (20%) Calcium oxalate monohydrate (20%) Proteins (15%) Glycosaminoglycans (15%)	Apatite Calcium oxalate dihydrate Calcium oxalate monohydrate
12	Calcium oxalate monohydrate (80%) N-Acetyl-Sulfamethoxazole (20%)	Calcium oxalate monohydrate N-Acetyl-Sulfamethoxazole
13	Calcium oxalate monohydrate (100%) Triamterene and metabolites (traces)	Calcium oxalate monohydrate Apatite
14	Calcium oxalate monohydrate (70%) Atazanavir (30%).	Calcium oxalate monohydrate Atazanavir

Struvite: magnesium ammonium phosphate (triple phosphate)

**Fig 1:**
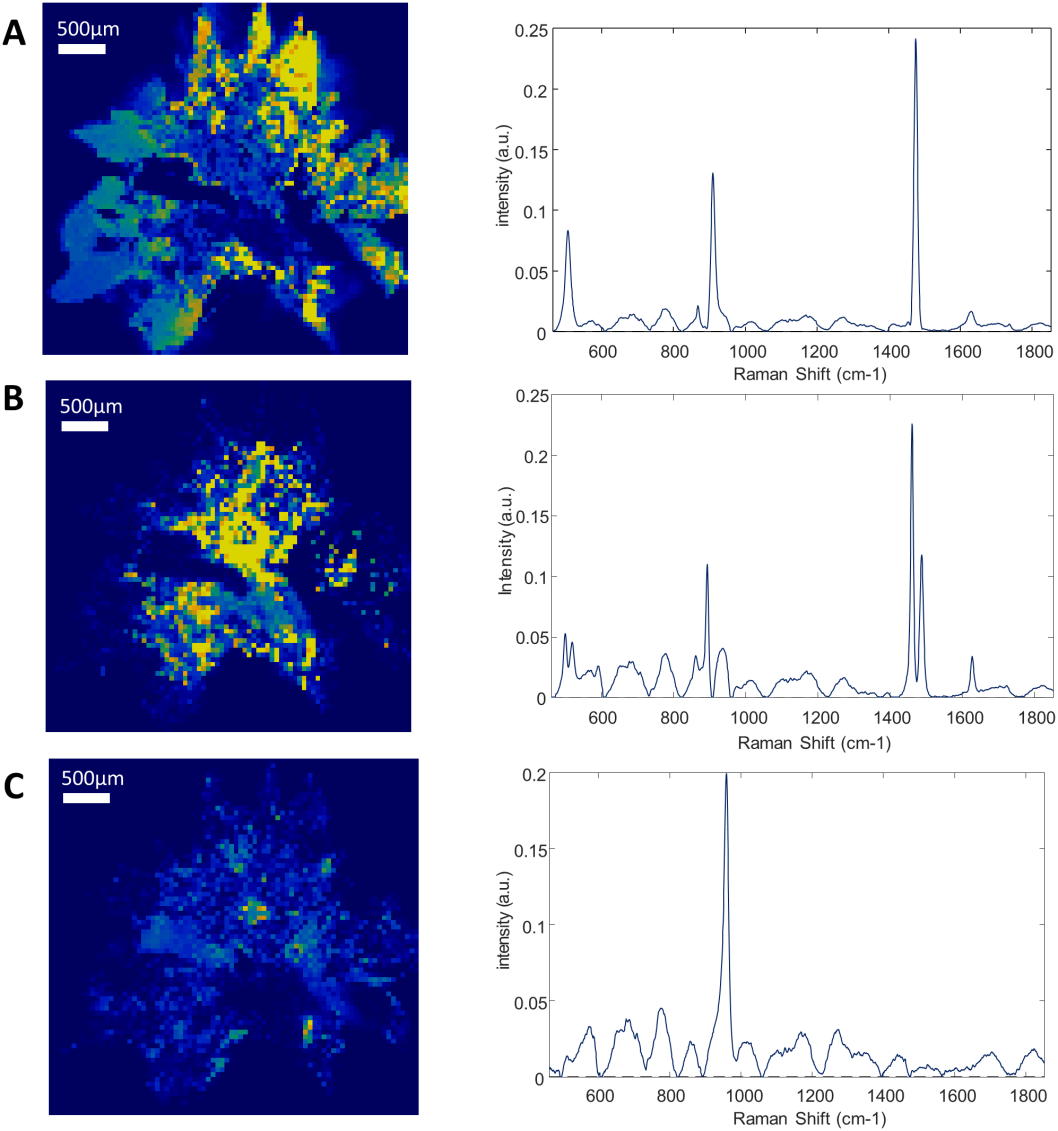
Raman mapping of sample 3. Raman mapping of the calcium oxalate dihydrate (A), calcium oxalate monohydrate (B) and apatite (C) and their correspondent Raman spectra. Step size: 50 μm. The yellow to dark blue scale represents the high density of a component or its absence. The peripheral spikes of the stones are mainly made of calcium oxalate dihydrate, while its center has turned into calcium oxalate monohydrate.

**Fig 2:**
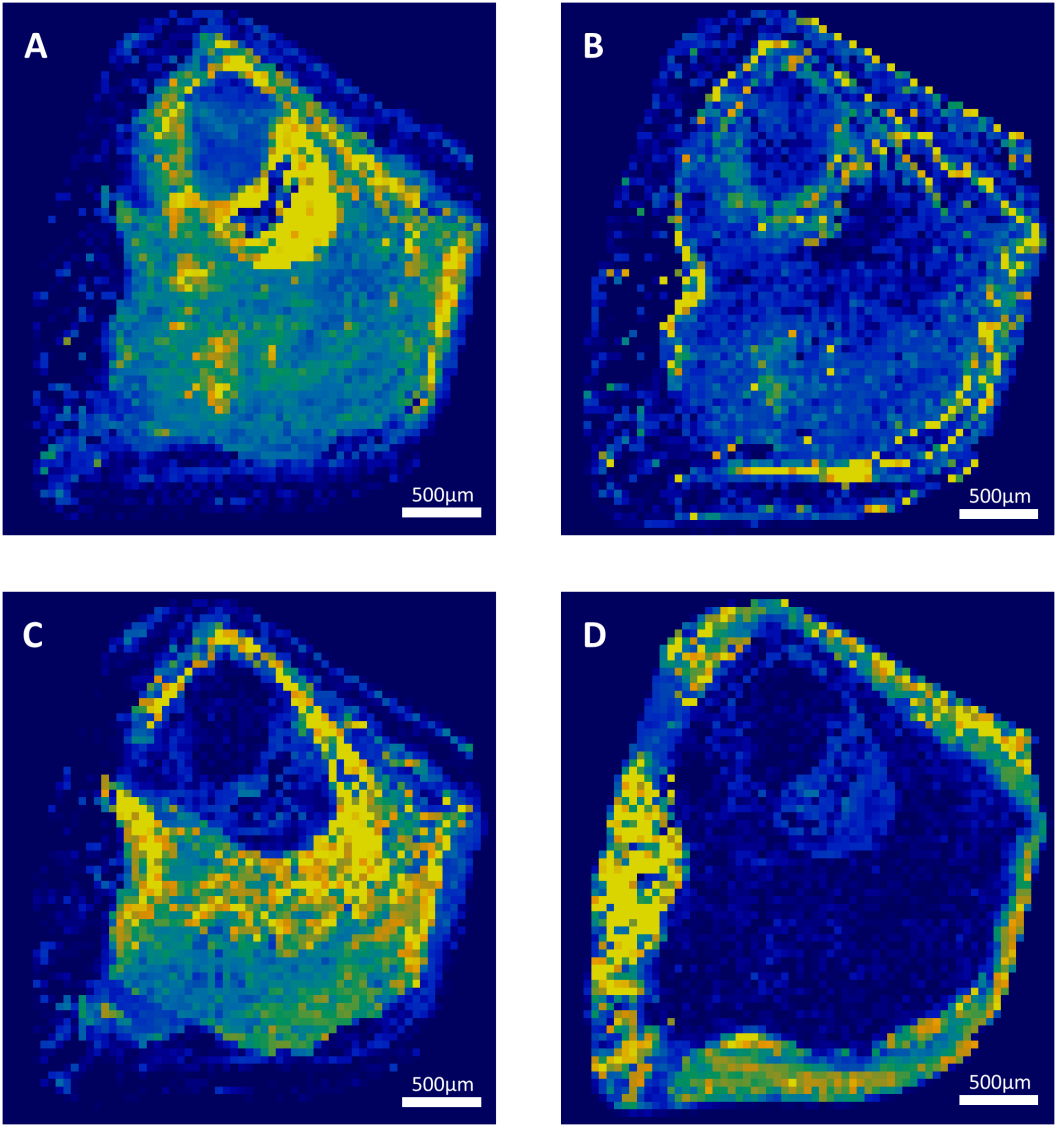
Raman mapping of sample 9. Raman mapping of whitlockite (A), calcium oxalate dihydrate (B), apatite (C), and brushite (D). Step size: 50 μm. The yellow to dark blue scale represents the high density of a component or its absence. The core of the stone was absent and provided low signal. The rest of the stone was made of whitlockite and apatite, probably due to former urinary tract infection, while the brushite coat is associated to recent hypercalciuria.

**Fig 3:**
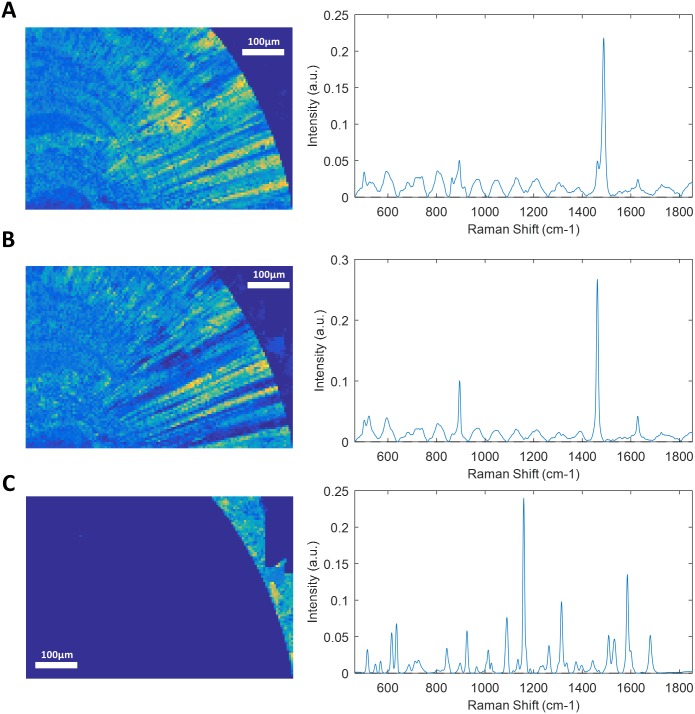
Raman mapping of sample 12. Raman mapping of calcium oxalate monohydrate with main absorption peak at 1487 (A) or at 1462 (B) and N-Acetyl-Sulfamethoxazole external deposit (C) and their correspondent Raman spectra. Step size: 5μm. The yellow to dark blue scale represents the high density of a component or its absence. The difference in main absorption peak of calcium oxalate monohydrate reveals a radial structure of the stone.

Each component correctly identified by RCI was also precisely localized in a 2-dimensions map representation of the sliced face of the stone. Successive layers of different compositions were observed within several stones. The sample 3 is mostly composed of calcium oxalate. However, there is a polarized distribution of calcium oxalate monohydrate and dihydrate within the stone (Fig 1). Indeed, the stone’s core is made of calcium oxalate monohydrate, while the sharp external crystals are essentially composed of calcium oxalate dihydrate. In the same way, the localization of the sample 9 nucleus was clearly localized (Fig 2). However, it was constituted of a gap that didn’t provide a strong Raman spectrum. This was confirmed by topological analysis (S2 Fig). The core was then largely covered of whitlockite mixed with apatite. Finally, a thin layer of COD lies beneath an external brushite coat. The nuclei of these two samples were precisely defined. In the sample 12, the thin layer of N-Acetyl-Sulfamethoxazole (around 100μm) was distinguished from the COM core (Fig 3). Interestingly, the COM had different Raman spectra according to the stone structure. The usual spectrum of COM presents a main absorption peak at 1487 or 1462cm^-1^. After mathematical manipulation of spectra, we were able to identify radial regions of the stones with higher absorption intensity peak at one of these two wavelengths (Fig 3A and 3B). In the three samples, the topological analysis allowed to easily identify regions where lower spectrum intensity was related to porosities (S1–S3 Figs).

## Discussion

### Identification of components

We report in this paper that RCI is a relevant method for kidney stone analysis. We tested in this study a large number of kidney stone components. The following constituents were correctly identified by RCI: COM, COD, anhydrous uric acid, uric acid dihydrate, ammonium urate, apatite, magnesium ammonium phosphate, brushite and whitlockite. These findings are consistent with others previous reports using conventional Raman spectroscopy [17,25]. The main Raman vibrational bands of common compounds have been described by Raman spectroscopy, but without providing samples mapping [16,17,20]. These compounds are the most commonly found in kidney stones but other constituents, even if they are less frequent, are important for stone-formers management [8]. In addition, cystine, xanthine and 2,8-dihydroxyadenine, three urolithiasis appearing in some hereditary disease, have also been identified by Raman spectroscopy [17,23]. It is mandatory not to miss these compounds because they are associated with very rare but life-threatening diseases requiring specific treatment [10].

Drugs crystals, which are often found in the urine of treated patients, are also susceptible to form kidney stones. The identification of a drug or of its metabolites in a stone leads directly to specific recommendations (hydratation or even stop drug intake). While Hong *et al* reported a sample of triamterene stone, no other drug calculi has been analyzed since by Raman vibrational method, to our knowledge [17]. We failed to measure Triamterene and its metabolites in sample 13, probably because it was present in traces at the surface of the stone and because it was too crumbly to resist to the milling process. However, RCI managed to localize N-Acetyl-Sulfamethoxazole and Atazanavir layers in two other samples, while these compounds hadn’t been described yet in urinary stones to our knowledge. Raman spectrometry has been widely used in drug quality control, and so, wide database of drugs spectra are available for their assessment [40]. However, the calculi may also contain drugs metabolites, like we showed it with N-Acetyl-Sulfamethoxazole and sulfate and hydroxyl conjugates of Triamterene. The spectra of those metabolites may not be available in databases. In other studies, Raman micro spectroscopy succeeded in providing the cellular distribution of drugs like doxorubicin or erlotinib [41,42]. Other studies showed that the identification of drugs within tissue could also be assessed [43]. In example, biphosphonate measurement was assessed within a 20mm thick porcine bone, which is a matrix similar to kidney stone [44].

Proteins and glycosaminoglycans could not be detected in our samples (samples 2, 7, 9 and 11), even in high quantity, because of the huge autofluorescence background of these poor Raman scatterers. Proteins are always present in urinary calculi, but usually in traces. When their proportion is higher, they might be associated to urinary tract infection, urothelial or glomerular damage. This is particularly challenging in case of infectious and drug induced stones, which often contain high organic matrix [45].

Raman spectroscopic analyses have also been used for distinction of polymorphic forms of crystals in drug industry [40]. Crystal’s polymorphism is important in stones determination for distinction between COM and COD, anhydrous uric acid and uric acid dihydrate, or between the different calcium phosphates. In example, dihydrate polymorph of uric acid nephrolithiasis has been associated to lower recurrence rate and higher BMI than anhydrous uric acid ones [46]. In our study, RCI was able to distinguish all these polymorphs ones from each other.

Quantification of the components by RCI is feasible, even if it was not carried out in this study. An accurate quantification may be used for instance to calculate the ratio of apatite to calcium oxalate for the screening of patients with hyperparathyroidism [47]. Yet, the quantification is still an issue, especially when automated analysis is performed by a software with a library comparison [17]. Raman and FTIR spectroscopy provide spectra of the whole sample which can be analyzed by the software [48]. However the library compares it to the best hits, and identifies a mix of up to three components maximum. The RCI increases the detection rate of small elements of interest. In 2013 Miernik et al developed a homemade Raman device for the analysis of freshly extracted renal stones [23]. It was used to analyze samples at 6 random spots on the surface without preparation (nor slicing or drying). The method showed a good sensitivity and specificity, but components of less than 25% may not be detected due to the random targeting. Nevertheless we believe this not accurate enough for a quality stone analysis. Actually, it is mandatory not to miss some constituents that are associated to specific and severe conditions, even when they are in small quantity, such as Cystine or Struvite in lysine-cystinuria and urease-producing urinary tract infection. We showed that RCI with a resolution of 50μm was able to discriminate up to 4 different components within a single sample, which is particularly useful in the highly complex stones that we selected. In addition, apatite and calcium oxalate were identified even when they represented less than 5% of stones, and RCI also identified apatite deposits that couldn’t be detected by FTIR in a sample.

### Stone structure and morphology

In addition to stone composition, RCI provided a precise mapping of components’ distribution within samples. The nuclei were accurately localized, as well as thin layers of other components. The morphology of the stones and succession of different layers can reliably inform about the “history” of stone formation. The majority of human urinary calculi are heterogeneous and are made of at least 2 different elements [48–50]. Kidney stones have various morphology and the distribution of components within their section is highly variable and complex [51]. Composition can vary significantly between surface, inner layers and core, especially for calcium oxalate and calcium phosphates stones. Calcium phosphates are more frequently present in the core of stones than in the external layers. Hence, the stone core may be associated to different urine sursaturation risk than the external and more recent layers. The treatment of nephrolithiasis patients should take this in consideration.

The different components are associated to different urine biochemical perturbations and different pathologies. The COD stones are mainly associated to hypercalciuria [18,52]. The mixed calculi of COD with apatite are associated to hypercalciuria rather than hypocitraturia in pure apatite stones [52]. A calcium oxalate stone with apatite core may have been initiated by an urinary tract infection [50]. The Daudon team have extensively studied stone structure and related it to specific conditions [53,54]. Hyperparathyroidism was more frequently associated to stones with specific morphology called IVd or II/IVa1 [47]. These stones are made of brushite or of COD and apatite layers. For more details about stone morphology classification, please refer to Daudon et al [54]. Very small spots of apatite smaller than 0.5mm on the surface of some COM stones attached to renal papilla are known as Randall’s plaque [28]. These patients may have altered gene expression associated to renal inflammation and oxidative stress [55]. It is important to detect peculiar structures, despite that they represent only a tiny part of samples because they can be clinically relevant.

All these elements are arguments to get an accurate and precise analysis of stones integrating component’s localization.

In our study, the nucleus of the stone 9 was constituted of whitlockite, surrounded by apatite, and finally coated with brushite (Fig 2). We assume there was a change in urine composition that led to the precipitation of these different phases. The core of the stone may be associated to ancient recurrent urinary tract infection that has led to calcium phosphates precipitation. In contrast, we assume that the brushite and COD external crystals are linked to hypercalciuria at the time of stone removal. The treatment of this stone-former should follow calcium stone recommendation (including hyperparathyroidism screening) instead of antibiotherapy if other symptoms of urinary tract infection have ceased.

As it can be seen in sample 12 (Fig 3), COM present different spectra according to its localization within the stones. The intensity of absorbance peaks is variable across the stone section. After mathematical resolution of spectra, some COM spots had a main absorbance peak at 1487cm^-1^ (Fig 3A), and others at 1462cm^-1^ (Fig 3B). This variation of calcium oxalate spectrum was associated to the stone structure. The two types of COM spectra are organized in radial lines coming from the stone core. In most cases, the section of COM stones has radial structure that is observable with a microscope or with scanning electron microscopy [52,56]. In contrast, it has been suspected that the absence of radial organization in some COM stones could be associated to a COD polymorphic conversion, or to primary hyperoxaluria when stone is also very clear in color [56,57]. According to Daudon, the COM stones of type Ic (unorganized, light colour) have up to 82% of recurrence, compared to the type Ia (radial and darker) which has a lower rate of recurrence of 34% [58]. RCI provided an easiest and more standardized way to detect radial organization of COM and could decrease inter-observer variability.

We showed in the sample 3 that the external, recent, sharp crystals were composed of COD, whereas the older part of the stone, the centre, was made up of COM (Fig 1). We assume that through time, urine retention may have led to crystal conversion of COD into COM, which is the most stable thermodynamic form [59]. Furthermore, there was no radial organization of COM according to the spectra, at the opposite of sample 12. This also suggest that COM hasn’t grown as usual but rather results of a conversion of COD. Indeed, it has been showed that structure of calcium oxalate stones can be reliably correlated to their composition. Duan et al showed that COM stones could be accurately distinguished from COD stones according to their shape using a computerized tomography [60]. They developed an automated calculation of shape index from tomography results that showed more curves in COD stones. This index could be applied to estimate stone response to shock wave lithotripsy treatment. The COM and COD appear in different urine sursaturation conditions. While COM is preferentially produced in hyperoxaluric urines, COD precipitates when the ratio of urine oxalate to calcium is decreased [61,62]. Also, COD stone-formers are generally younger and have increased risk of stone recurrence [63,64]. These considerations are important for the treatment concerning calcium intake as well as resistance to shock-wave lithotripsy for example [60]. The internal deposits of apatite in this sample can be associated to a change in urine composition with hypercalciuria and hypocitraturia, rather than to urinary tract infection as mentioned above. Blanco et al also suggested that the urine could flow between the crystals of COD after their formation, where apatite would latter precipitated because of urinary stasis [65]. All these considerations imply different treatment and follow-up for the patient.

Furthermore, the good RCI resolution (lower than 2–3 μm) improved sensitivity and allowed the detection of very small elements like nuclei and Randall’s plaques, that are difficult to identify with FTIR or X-ray diffraction analysis. Randall’s plaques are small spots of apatite appearing on the renal papilla that acts as nucleation factors for stone growth. They are more and more frequently found on the top of COM stones [26]. In addition, they must be differentiated of random apatite deposits that simply precipitate when urine pH is higher. In both cases, FTIR or X-ray diffraction analyses will provide low apatite percentage, while RCI can distinguish them by their localization.

### Comparison to other methods

In this study, we compared RCI to FTIR spectroscopy in kidney stone analysis. The FTIR is the gold standard method, and powder X-Ray diffraction has also shown proof of performance in stone determination, but is less used [30,35,66]. Both of them are well documented for stone analysis, but they don’t provide any structural information compared to RCI. In several studies comparing Raman spectroscopy to FTIR, the analysis of samples revealed that some components were only identified by one of the two methods [23]. Raman was almost identical to FTIR for components identification, and could be even more potent in some cases [25].

More sophisticated methods, such as microCT-scan and electron-scanning microscopy have also been developed to gather extensive structural information. However, they are currently exclusively reserved for study purpose, because they’re particularly expensive and time-consuming. In addition they are not specific enough in discriminating some similar constituents [14,24,67]. These methods are more accurate for morphology determination than RCI, but are less precise in composition determination. In summary, all techniques present disadvantages, and thus, should be combined in order to avoid loss of information and to obtain an optimal comprehension of metabolic risk factors involved in lithogenic pathway. Compared to other methods, RCI presents a good combination between vibrational techniques and structure-oriented analyses that are very different [14,68]. The time of analysis for a single sample varied from 10 to 36 h. It is very time-consuming for clinical laboratory, and makes it more suitable for research studies rather than routine analysis. RCI is non-destructive, enabling the storage of samples.

Raman spectroscopy measures the scattering of vibrating and rotating molecules, while FTIR microscopy assess the absorption of these molecules. Both methods are similar and complementary, but each has advantages and drawbacks regarding the resolution of spectra and imaging. Raman requires little sample preparation, and isn’t impeded by water or urea, which is useful in urolithiasis analysis [17,23,69,70]. In addition, it is commonly accepted that Raman has a better spatial resolution, instead of a lower spectral resolution and longer analysis time compared to FTIR microscopy [31,69–72]. Also, the Raman source can be destructive, particularly with poor Raman scatterers like proteins.

Daudon et al used Raman spectroscopy associated to MOLE (laser molecular microsond) that can analyze very small crystals of 1 or 2μm [16]. More recently, Shameem et al have evaluated micro-Raman microscopy associated to LIBS (Laser Induced Breakdown Spectroscopy) in urinary calculi investigation [51]. These methods are complementary and can provide molecular and elemental data of the samples with high resolution. LIBS-Raman requires sample sections with physically uniformity over a relatively large area [51]. In addition, Raman alone wasn’t able to identify all samples, and results weren’t confirmed by FTIR gold standard method. Both MOLE and LIBS are expensive and not easily available methods compared to the current Raman microscopes available on the market. Once again, elements distribution wasn’t provided in these papers. Tang et al recently characterized *Mycobacterium* using a 1μm resolution RCI device [69]. The high resolution of RCI allowed this method to even provide maps of cellular distribution of drugs within cellular organites [41,42].

Krafft et al tested both Raman and FTIR imaging on colon tissue [71]. RCI was performed with a step size of 2.5 μm. The authors confirmed that RCI had a better spatial and spectral resolution but lower spectral quality and longer analysis time compared to FTIR microscopy. Notably, some papers have also used FTIR microscopy to measure microcrystals within kidney. Daudon et al identified calcium phosphate and calcium oxalate crystals deposit in renal tissue [73]. Crystal’s plugs measured around 50μm and were detected in tissue with 4μm section and with a resolution of 6.25μm. These drug deposits are smaller than those we detected in our samples. N-Acetyl-Sulfamethoxazole layer measured around 100μm of thickness in sample 12. Similarly, in a case-report, the authors used infrared microscopy to identify small vancomycin deposits (100-900nm) within the kidney of a patient with acute renal failure [74]. Finally, and more remarkably, Blanco et al recently tested infrared microscopy on urinary calculi and managed to obtain a resolution of 80μm providing an accurate mapping of stones components [65]. In a sample, they identified up to 5 different regions with different spectra, from the core to the surface. FTIR imaging resolution is limited by diffraction close to 25μm [72]. The resolution can be improved by using a combination with other techniques like synchrotron, atomic force microscopy or ATR. A resolution of 10μm could be obtained using a FTIR microscopy with a synchrotron source for crystals deposits in renal biopsy with metal coated glass [70]. Obviously, this kind of sample preparation isn’t suitable for renal stones samples that measure several millimeters and cannot be sliced at the μm range. Still, Raman microspectrosopy has better imaging resolution allowing the identification of cells and organites as mentioned above but is way more slower, which makes infrared microscopy a promising method [31,41,42].

### Bias of the study

The main bias of this study is that we did not perform quantification of components by RCI. It is also possible to distinguish highly from weakly carbonated apatite with FTIR analysis. Daudon et al also assessed this parameters with Raman microprobe [16]. This difference has not been recorded with RCI in this study, despite it is of interest for urinary tract infection screening [75]. Then, the samples could not be analyzed fully by both methods, and the method required the selection of different sample fragments of the same stone. RCI is not a destructive method but the glue used for analysis would have impaired the FTIR measurement. Despite we measured the most frequent stone’s components with RCI, we only assessed a small set of samples. Unfortunately, we have no FTIR microscope in our possession, and we could not directly compare it to RCI.

### Perspectives

Other human biological samples than kidney stones have also been assessed with Raman analysis. RCI has been widely tested in cancer diagnosis [31]. Actually, histological classification of cancers is mandatory in diagnosis and treatment [32,76]. Therefore, RCI can provide tumor tissue mapping that is useful for the management of cancers. Raman microscopy was able to discriminate normal tissue from low or high grade squamous intraepithelial lesion in cervical tissue [33]. In the study of Rashid et al, RCI also identified tissue layers with biochemical anomaly that weren’t noticed on photonic microscopy despite using a quite low spatial resolution of 100μm. RCI has even been used during brain surgery to differentiate tumor-infiltrated tissues from non-infiltrated tissues [77]. This method was highly correlated with histology results, and had a sensitivity and sensibility of 93% and 91% respectively with a resolution of 300 to 25μm. Some studies combined chemical and biological information to morphological and distribution parameters to be able to identify correctly tumors or subcellular organites with high accuracy, sometimes even better than classical histology [31].

Considering urolithiasis measurement is highly variable between laboratories and is analyst-dependant [35], such methods should be applicable to calculi assessment in order to decrease operator variability and to provide high quality analysis. Indeed, as demonstrated by two quality assessment of urinary stone analysis, the lack of education in recognizing interesting elements is a major concern, particularly for destructive methods [35,66]. The development of automated software analysis and computer aided diagnostic should decrease inter-observer and inter-laboratory variability [31,69].

Analysis of wet sample was possible in classical Raman spectroscopy [78] but may be a limitation in RCI because of the slicing would not be regular. When the sliced face is not smooth enough, porosities give a weaker signal. This is also an issue with some chemicals that are too crumbly, like apatite, MAP and proteins. This step can be overcome with 3D mapping devices that allow to maintain focus on curved or rough surfaces, without further sample preparation [79,80]. In addition, it could easily give information about crystals geometry or surface as it has been shown on bone or cartilage, as well as with rocks. However, in our study, the topological analysis was of limited use because the samples were milled according to our protocol.

## Conclusion

It is the first time the Raman chemical imaging is used in order to produce 2D map of kidney stones. We showed that RCI is an efficient tool to identify kidney stone components in comparison to the FTIR gold standard analysis. Moreover, RCI provided useful distribution mapping of constituents that allow a better detection of small structures like nuclei and drug layers, increasing sensitivity. RCI allowed detecting COD conversion to COM, and radial structure of COM stones. This analysis is semi-destructive but time-consuming. The analysis was a good compromise between FTIR and other advanced technologies like micro-CTscan, but might be exceeded by infrared microscopy. Proteins measurement is still an issue that we didn’t manage to overcome and that may be troublesome in quality assessment.

## Supporting information

S1 Fig Topological analysis of sample 3. Gaps provide grey to black spots according to their depth, while the whiter zones are associated with more intense Raman signal.
(TIF)

S2 Fig Topological analysis of sample 9. Gaps provide grey to black spots according to their depth, while the whiter zones are associated with more intense Raman signal.
(TIF)

S3 Fig Topological analysis of sample 12. Gaps provide grey to black spots according to their depth, while the whiter zones are associated with more intense Raman signal.
(TIF)

## Abbreviations

COD: Calcium oxalate dihydrate
COM: Calcium oxalate monohydrate
FTIR: Fourier Transform Infrared spectroscopy
RCI: Raman chemical imaging
