# Supplementary figures and images for: Raman chemical imaging, a new tool in kidney stone structure analysis: Case-study and comparison to Fourier Transform Infrared spectroscopy
Source: PLoS One. 2018 Aug 3;13(8):e0201460. doi: 10.1371/journal.pone.0201460 (PMC6075768; doi:10.1371/journal.pone.0201460)

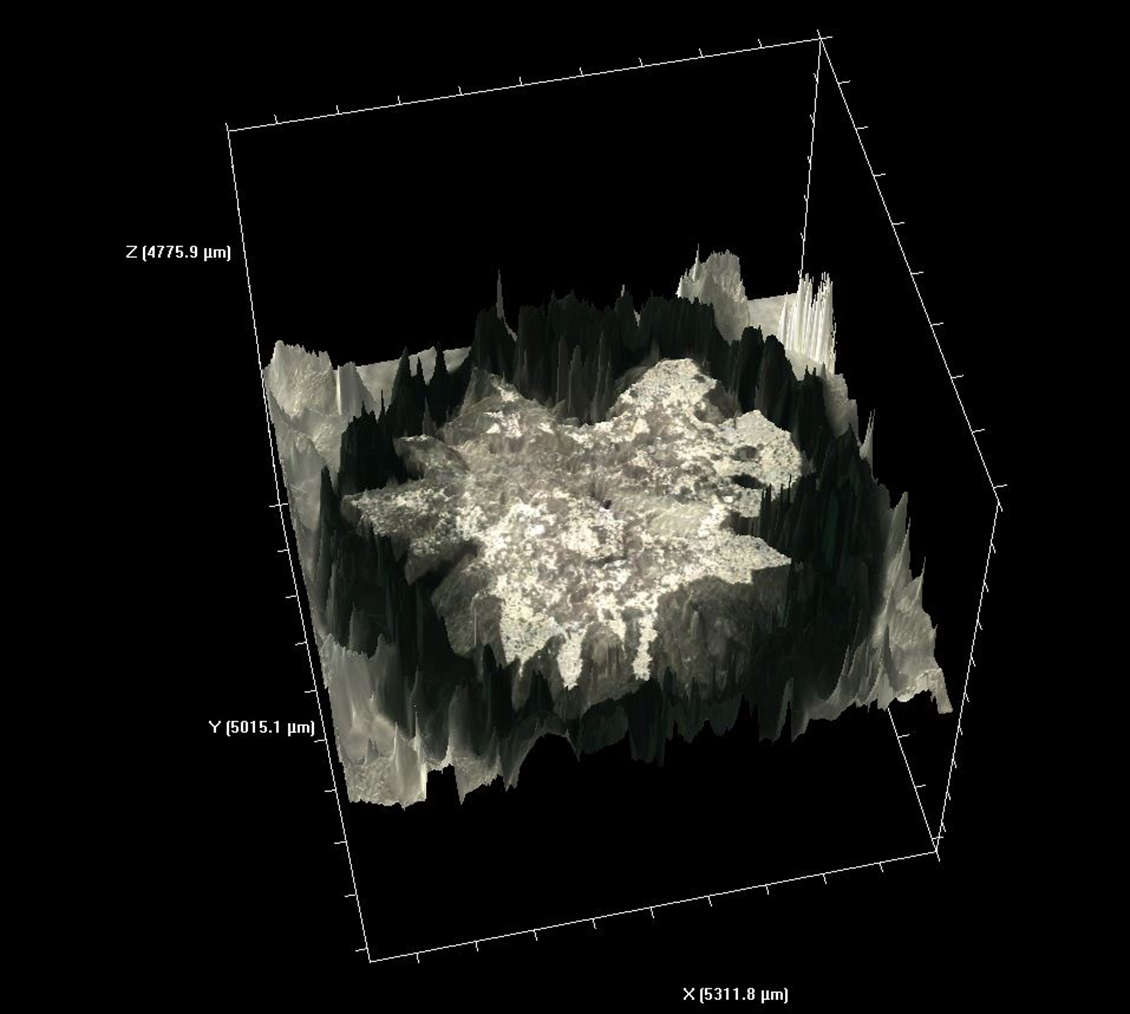

Supplement: S1 Fig — Gaps provide grey to black spots according to their depth, while the whiter zones are associated with more intense Raman signal. (TIF) [file pone.0201460.s001.tif]

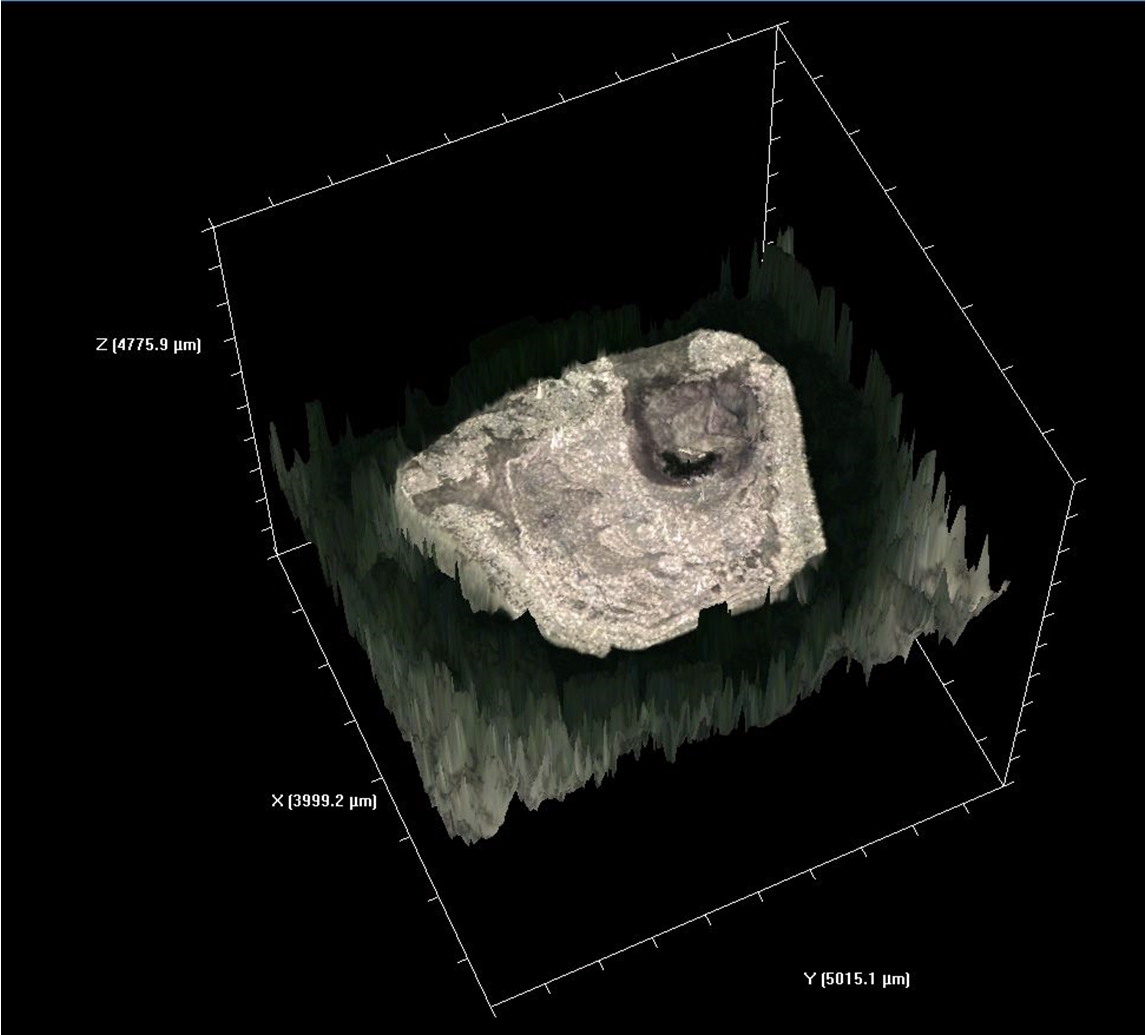

Supplement: S2 Fig — Gaps provide grey to black spots according to their depth, while the whiter zones are associated with more intense Raman signal. (TIF) [file pone.0201460.s002.tif]

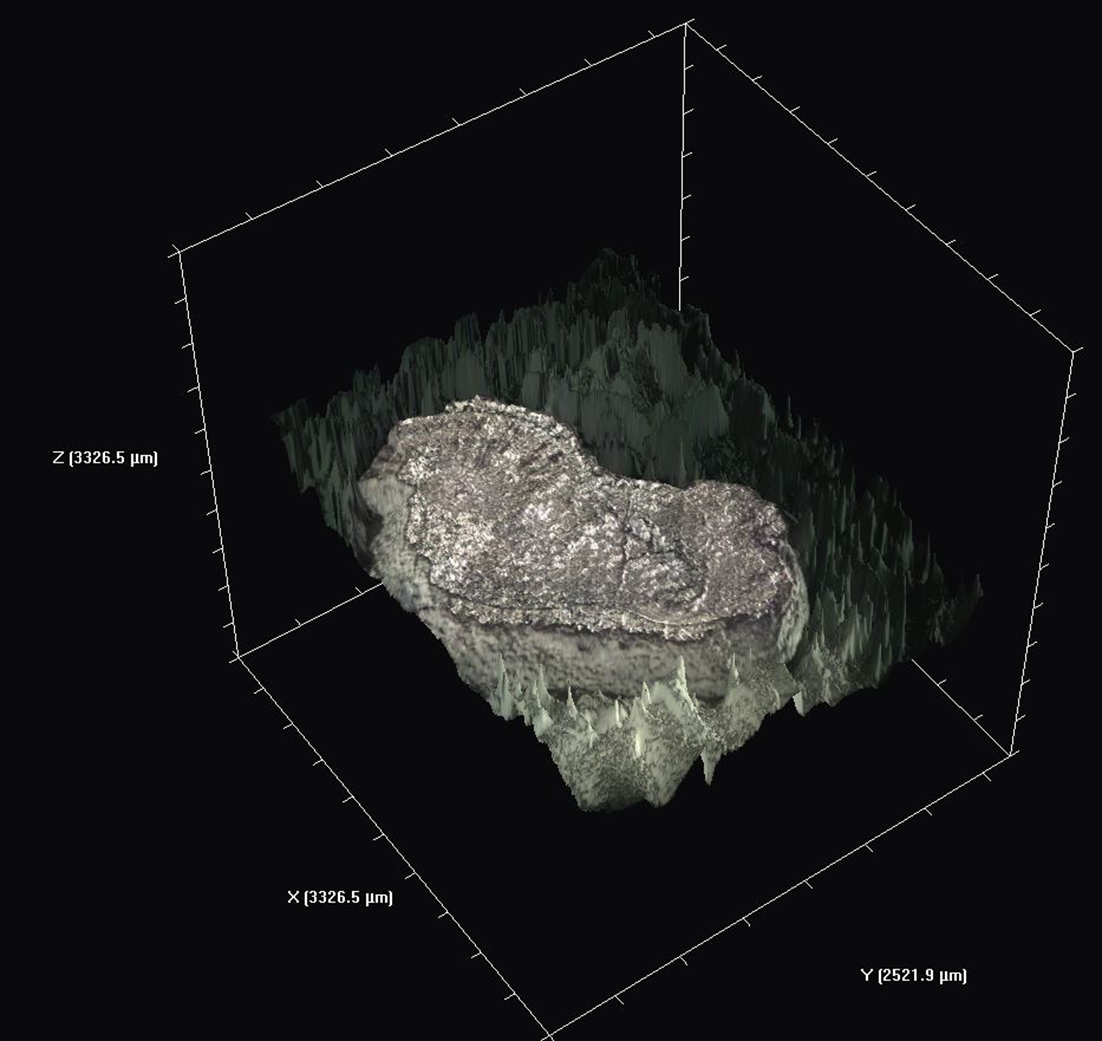

Supplement: S3 Fig — Gaps provide grey to black spots according to their depth, while the whiter zones are associated with more intense Raman signal. (TIF) [file pone.0201460.s003.tif]
